# VEGF‐A Antisense Oligonucleotide Therapy Inhibits Corneal Neovascularisation and Enhances Graft Survival in Murine High‐Risk Corneal Transplantation

**DOI:** 10.1111/ceo.70159

**Published:** 2026-09-09

**Authors:** Wei Zhang, Christian Weile, Christoph Ullmer, Sascha Fauser, Felix Bock, Claus Cursiefen

**Affiliations:** ^1^ Department of Ophthalmology University of Cologne, Faculty of Medicine and University Hospital Cologne Cologne Germany; ^2^ Department of Ophthalmology, Hebei Eye Hospital Hebei Key Laboratory of Ophthalmology Xingtai Hebei China; ^3^ Department of Ophthalmology Affiliated Hospital of Guizhou Medical University, Guizhou Medical University Guiyang China; ^4^ BioInnovation Institute Copenhagen Denmark; ^5^ Roche Pharma Research & Early Development Roche Innovation Center Basel, F. Hoffmann‐La Roche Ltd Basel Switzerland; ^6^ Center for Molecular Medicine Cologne (CMMC) University of Cologne Cologne Germany; ^7^ CECAD Cluster of Excellence University of Cologne Cologne Germany

## Abstract

**Background:**

To investigate, for the first time, whether an unconjugated VEGF‐A antisense oligonucleotide (ASO) can inhibit corneal neovascularisation and improve graft survival in a murine high‐risk corneal transplantation model.

**Methods:**

A suture‐induced corneal neovascularisation model was established in BALB/c mice, followed by high‐risk corneal transplantation using C57BL/6 donor corneas. A VEGF‐A RNase H–recruiting ASO (gapmer) was delivered via subconjunctival injection, topical eye drops, or ex vivo preincubation of donor corneas. Blood vessels (BVs) and lymphatic vessels (LVs) were quantified by immunohistochemistry, and *Vegfa* mRNA expression was measured by qPCR. Immune cell profiles in draining lymph nodes (dLNs), focusing on regulatory T cells (Tregs) and dendritic cells (DCs), were analysed by flow cytometry. Graft survival was monitored for 8 weeks using Kaplan–Meier analysis.

**Results:**

Subconjunctival and topical VEGF‐A ASO administration significantly suppressed corneal hemangiogenesis and lymphangiogenesis (*p* < 0.05). Subconjunctival injection also induced regression of established vessels (BVs −21.76%, *p* = 0.0039; LVs −49.75%, *p* = 0.0110). Graft survival improved significantly after subconjunctival treatment (*p* = 0.0438) and donor cornea preincubation (*p* = 0.0148). Flow cytometry showed increased Treg frequency in dLNs following subconjunctival ASO administration (*p* = 0.0293). Preincubation of donor corneas markedly reduced *Vegfa* mRNA levels (*p* < 0.0001).

**Conclusions:**

VEGF‐A–targeting ASO effectively inhibits corneal neovascularisation and improves graft survival in high‐risk transplantation. Local and ex vivo VEGF‐A inhibition using ASO represents a promising therapeutic strategy for controlling ocular neovascularisation and enhancing corneal transplant outcomes.

## 1 Introduction

Corneal neovascularisation, that is, the pathologic ingrowths of blood (BVs) and lymphatic vessels (LVs) into the normally avascular cornea, endangers vision and leads to increased graft rejections after therapeutic corneal transplantation. Corneal transplantation is the most frequently performed tissue transplantation [1]. However, immune‐mediated rejection remains a significant barrier to long‐term graft survival, particularly in high‐risk recipients with pre‐existing corneal neovascularisation [2, 3]. The pathological growth of blood (hemangiogenesis) and lymphatic (lymphangiogenesis) vessels facilitates antigen‐presenting cell migration and alloimmune sensitisation, increasing the risk of graft failure despite systemic immunosuppressive therapy [4]. Among the key mediators of this process, VEGF‐A plays a crucial role in promoting both blood and lymphatic vessel formation, making it a prime target for therapeutic intervention [5, 6, 7, 8]. The potential of targeting VEGF‐A to promote high‐risk graft survival has been shown [5, 9].

Emerging RNA‐targeting therapies provide a strategy for targeting VEGF‐A at the mRNA level, enabling more prolonged and precise inhibition of pathological neovascularisation [10, 11]. Compared to monoclonal antibodies, RNAse H‐recruiting antisense oligonucleotides (ASOs) exhibit superior stability and the potential for local delivery without the need for a carrier [12, 13, 14]. These properties make VEGF‐A ASOs a promising approach for sustained suppression of corneal neovascularisation. Given the eye's accessibility, local administration of VEGF‐A ASOs via topical eye drops, subconjunctival injection, or donor cornea preincubation provides a targeted and minimally invasive therapeutic strategy to improve transplant outcomes. However, VEGF‐A ASOs have not been tested in the cornea in this way.

This study investigates for the first time the therapeutic potential of a VEGF‐A ASO in inhibiting corneal neovascularisation and enhancing graft survival in high‐risk keratoplasty. The findings from this study offer new insights into RNA‐targeted VEGF‐A suppression as a viable strategy for inhibiting vision threatening corneal neovascularisation [15, 16, 17, 18] and for improving corneal transplant success and may have broader implications for other ocular and neovascularisation‐induced disorders such as wet age‐related macular degeneration (AMD) and diabetic retinopathy.

## 2 Methods

### 2.1 Animals and Anaesthesia

All animal experiments were conducted in accordance with the ARVO Statement for the Use of Animals in Ophthalmic and Vision Research. The study was reviewed and approved by the State Agency for Nature, Environment and Consumer Protection of North Rhine‐Westphalia (LANUV NRW; approval no. TVA 81‐02.04.2021. A214). Female BALB/c mice (6–8 weeks old) obtained from Charles River Laboratories (Sulzfeld, Germany) were utilised for the suture‐induced corneal neovascularisation model. For corneal transplantation, age‐matched female C57BL/6 mice served as donors, while BALB/c mice were recipients. Anaesthesia was administered via intraperitoneal injection of Ketanest (8 mg/kg) and Rompun (0.1 mL/kg) before surgery.

### 2.2 Suture‐Induced Inflammatory Corneal Neovascularisation

The mouse model for suture‐induced corneal inflammation and neovascularisation was implemented following established protocols [9]. Three 11‐0 nylon sutures (Serag Wiessner, Naila, Germany) were introduced into the corneal stroma of the right eye, with each suture forming two stromal penetrations, covering approximately 120 degrees of the corneal circumference. The sutures were positioned with the outer anchoring points near the limbus, while the inner placements were equidistant between the limbus and the central cornea to achieve a consistent angiogenic response (Figure 1A).

**FIGURE 1:**
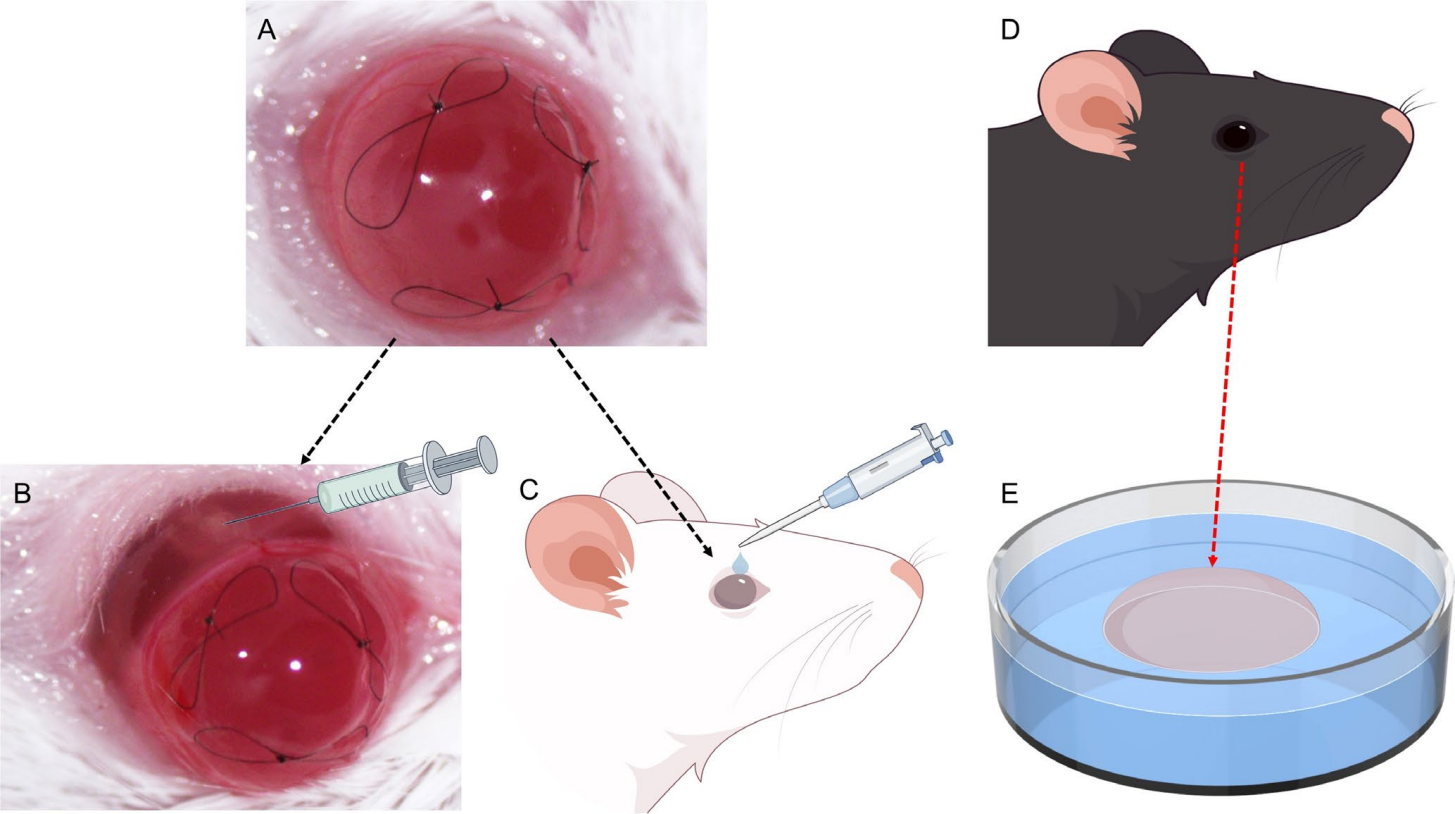
Experimental model and treatment approaches using a VEGF‐A ASO. (A) Three figure‐eight intrastromal sutures were placed in right cornea of BALB/c mice to induce corneal neovascularisation. (B) Subconjunctival injection of VEGF‐A ASO or non‐targeting ASO was performed 1 mm posterior to the limbus. (C) Topical application of VEGF‐A ASO or non‐targeting ASO via eye drops in the same model. (D, E) Preincubation of donor corneas from C57/BL6 mice with VEGF‐A ASO or non‐targeting ASO prior to corneal transplantation in the murine model (by Figdraw). ASO, antisense oligonucleotide.

### 2.3 Treatment Administration

All in vivo procedures were performed unilaterally in the right eye. The contralateral left eye was left untreated and was not included in the analysis. An overview of eye assignment, suture placement, treatment allocation and corresponding figure panels is provided in Table S1.

For subconjunctival injection, 10 μL of the VEGF‐A ASO or a non‐targeting ASO (5 μg/μL, Roche, Switzerland) was administered into the right eye using a 32‐gauge syringe (Hamilton, Reno, Nevada, USA), with the injection site positioned 1 mm posterior to the limbus [11] (Figure 1B). The ASO was designed as a 16‐mer gapmer with phosphorothioate backbone, locked nucleic acid (LNA)‐modified nucleotides at both ends and a central DNA gap (Roche, Switzerland). For compound exposure and *Vegfa* mRNA analysis after subconjunctival delivery, injections were performed every other day after suture placement until tissue collection. For assessment of corneal neovascularisation inhibition, subconjunctival injections were administered after suture placement. For the regression model, treatment was initiated after corneal vessels maturation and sutures removal. In the high‐risk corneal transplantation model with postoperative treatment, VEGF‐A ASO or non‐targeting ASO was injected five times every other day subconjunctival into the right eye of the recipient after transplantation.

For topical application, 3 μL of the VEGF‐A ASO or a non‐targeting ASO control (5 μg/μL) was applied directly onto the corneal surface using a micropipette [19] (Figure 1B). Repeated drug application was performed throughout the respective experimental periods. For compound exposure and *Vegfa* mRNA analyses, eye drops were administered three times daily starting on Day 0 until tissue collection in both naïve and suture‐induced eyes. For assessment of corneal neovascularisation inhibition, eye drops were administered three times daily for seven consecutive days following suture placement.

For preincubation of corneal grafts, 2 mm central corneal buttons from C57BL/6N mice (Figure 1D) were incubated for 48 h at 37°C with 5% CO_2_ in Culture Medium I (Biochrom, Germany) containing either VEGF‐A ASO or a non‐targeting ASO (5 μg/μL) with 2% fetal calf serum [20] (FCS; Biochrom, Germany) (Figure 1E). After preincubation, donor corneal buttons were either collected for compound exposure and *Vegf‐a* mRNA analyses or transplanted into suture‐induced high‐risk recipient beds. In this case, the compounds were administered only once via the donor tissue.

For murine allogeneic corneal transplantation, corneal allografting was performed using 2.0 mm donor corneas, either naïve or preincubated for 48 h, from C57BL/6 mice. These grafts were transplanted onto suture‐induced high‐risk graft beds in BALB/c recipients. The grafts were secured with eight interrupted 11‐0 nylon sutures, followed by antibiotic treatment and temporary tarsorrhaphy to protect the graft. Sutures were removed after 7 days, and graft opacity was monitored weekly for 8 weeks [21, 22]. Opacity was graded on a scale from 0 (clear) to +5 (maximum opacity), with grafts scoring > 2 considered rejected [21, 23]. Cases with infection, abnormal graft presentation, or anterior chamber loss were excluded from the analysis.

### 2.4 Tissue Collection and Processing for Compound Exposure and Gene Expression Analysis

For subconjunctival injection, treatment was administered on every other day following suture placement (Figure 2A). For topical application (eye drops), the agent was applied three times daily to both suture‐induced and naïve corneas (Figure 2B). For corneal graft preincubation, 2 mm central corneal buttons were incubated with the designated agents for 48 h (Figure 2C). At the end of each experiment, collected corneas—including those with limbus tissue, conjunctiva and preincubated grafts—were snap frozen in liquid nitrogen for subsequent analysis.

**FIGURE 2:**
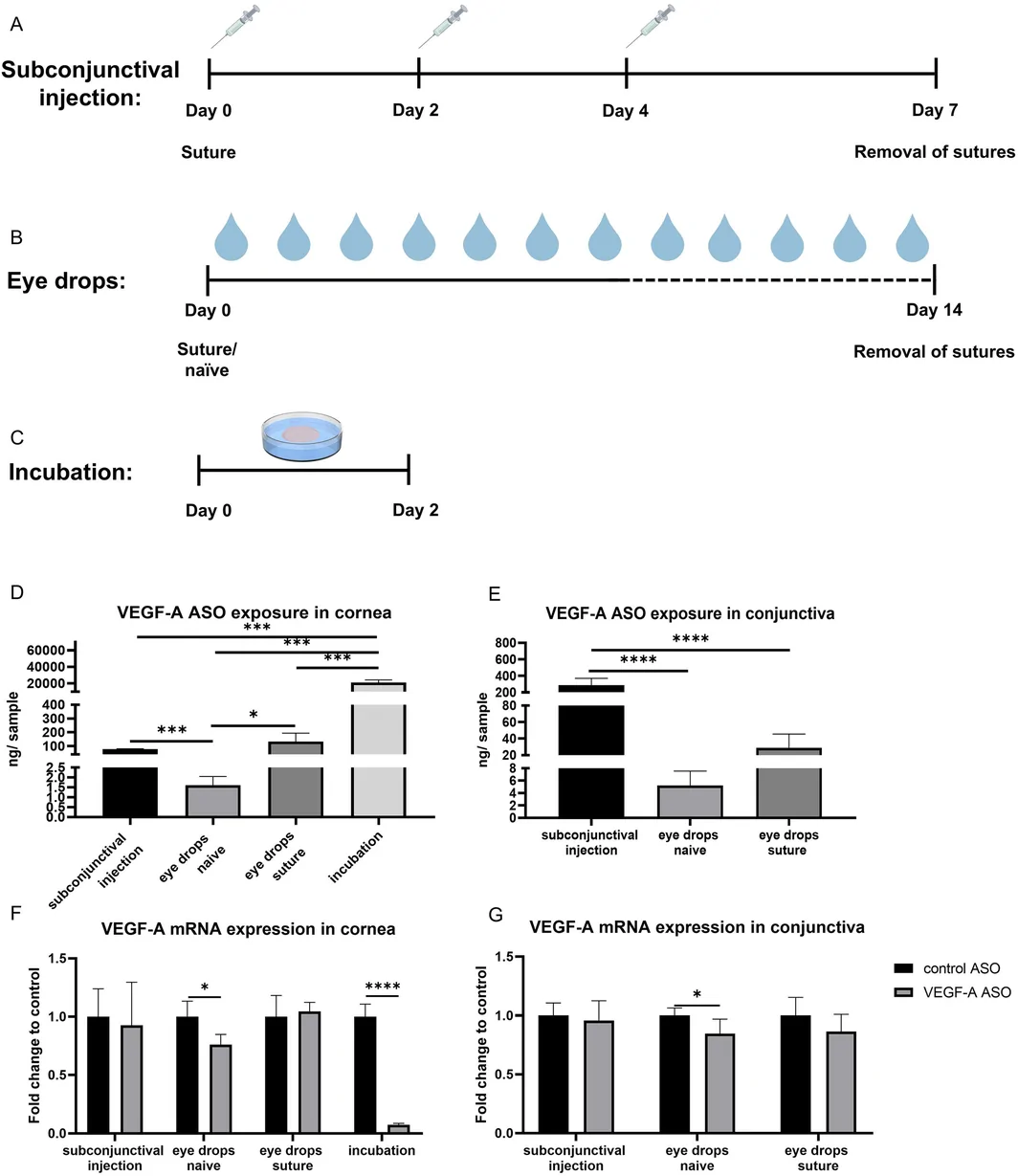
VEGF A ASO exposure analysis and relative *Vegfa* mRNA expression after different delivery approaches. (A) Experimental timeline for subconjunctival injection in the suture‐induced corneal neovascularisation model. VEGF‐A ASO or non‐targeting ASO was administered into the right eye every other day on Days 0, 2 and 4 after suture placement, and tissues were collected for analysis. (B) Eye drops were applied three times daily from Day 0 until tissue collection in both naïve and suture‐induced corneas, with suture removal on Day 14. (C) Preincubation of donor corneas with VEGF‐A ASO or non‐targeting occurred for 48 h. (D, E) After the above‐described treatment administrations, quantification of VEGF A ASO exposure in the cornea (D) and conjunctiva (E) across different treatment groups was performed. Preincubation resulted in the highest drug concentration in the cornea, while subconjunctival injection led to the highest exposure in the conjunctiva. Subconjunctival injection and eye drops (suture model) achieved moderate levels. Naïve eye drop application resulted in the lowest compound uptake. (F, G) Relative *Vegfa* mRNA expression in the cornea (F) and conjunctiva (G). A significant reduction in *Vegfa* mRNA levels was quantified in the cornea after preincubation and eye drop treatment of naïve eyes. A similar trend was found in the conjunctiva. No significant suppression was observed following subconjunctival injection or eye drop application in inflamed, vascularised corneas. *n* = 5 per group, *p < 0.05, ****p < 0.0001.

Frozen tissues were homogenised in 500 μL MagNA Pure Tissue lysis buffer (MagNA Pure 96 LC RNA Isolation Tissue, Roche) using a TissueLyser II (Qiagen; one stainless‐steel bead; 2 × 1.5 min at 30 Hz), incubated 30 min at room temperature to complete lysis, and split for ASO exposure measurement and RNA extraction (MagNA Pure 96, Roche).

### 2.5 ASO Exposure Quantification by Hybridisation ELISA

ASO levels were quantified using a two‐probe hybridisation enzyme‐linked immunosorbent assay (hELISA). Full‐locked LNA biotin‐ and digoxigenin‐labelled probes complementary to opposite ASO termini (35 nM each) were hybridised with samples in 5× saline‐sodium citrate (SSC) + 0.05% Tween‐20 for 30 min at room temperature in non‐binding plates (Nunc). Complexes were transferred to streptavidin‐coated plates (Roche; 1 h), washed 3× with 2× SSC + 0.05% Tween‐20, incubated with anti‐DIG–alkaline phosphatase Fab (Roche; 1:4000 in 5× SSCT; 1 h), washed 3×, developed with BluePhos substrate (KPL/SeraCare, Gaithersburg, MD, USA), and read at A615 nm (BioTek). Concentrations were interpolated from a standard curve generated by spiking the identical ASO into buffer (no untreated tissues available).

### 2.6 Gene Expression Analysis via RT‐qPCR

RNA extracted on the MagNA Pure 96 was assessed for yield/quality, diluted to 10 ng/μL, heat‐shocked for 40s at 90°C, and placed on ice. One‐step reverse transcription quantitative polymerase chain reaction (RT‐qPCR) was performed using qScript XLT One‐Step RT‐qPCR ToughMix, low ROX (QuantaBio) on a ViiA 7 instrument (Thermo Fisher) with single‐plex assays for mouse *Vegfa* (IDT, Mn.PT.58.14200306), Ppia (IDT, Mn.PT.39a.2.gs), and Hprt (IDT, Mn.PT.58.32092191). Each reaction used 10 ng RNA and was quantified against a standard curve generated from study‐sample RNA. *Vegfa* quantities were normalised to both Ppia and Hprt; the average of the two normalisers was used as the final expression value.

### 2.7 Immunohistochemistry and Morphological Analysis of Corneal Flat Mounts

Corneal whole mounts were subjected to double immunostaining to visualise BVs and LVs, following previously established protocols [9, 23, 24, 25]. Excised corneas were rinsed twice in PBS and fixed in acetone for 25 min. After three additional PBS washes, samples were blocked using 2% bovine serum albumin (BSA) in PBS, followed by overnight incubation at 4°C in the dark with primary antibodies: rabbit anti‐mouse LYVE‐1 (1:200; AngioBio, Del Mar, CA, USA) for LVs and FITC‐conjugated rat anti‐mouse CD31 (1:100; BD Pharmingen, BD Biosciences, USA) for BVs. The next day, LYVE‐1 labelling was detected using a goat anti‐rabbit Cy3‐conjugated secondary antibody (1:100; Dianova, Germany).

To capture whole mount images, 9–12 overlapping fields at 100× magnification were automatically assembled using a BX53 fluorescence microscope (Olympus Optical Co., Hamburg, Germany). Vessel coverage was quantified using an image analysis algorithm in Cell^F (Olympus Soft Imaging Solutions GmbH, Münster, Germany), as previously reported [23]. Briefly, grey‐scale images were processed using four sequential software‐based filters before analysis. The total corneal area was defined using the innermost vessel of the limbal arcade as the border (region of interest, ROI), and the vascularised area was calculated in mm^2^ based on a defined threshold [23]. To standardise comparisons, the mean vascularised area in the control ASO group was set as 100%, and vascularised areas in the treatment group were expressed relative to this reference.

### 2.8 Flow Cytometry Analysis of Ipsilateral Submandibular Lymph Nodes

Ipsilateral submandibular draining lymph nodes (dLNs) were harvested 8 weeks post‐keratoplasty. Single‐cell suspensions were prepared and stained with fluorochrome‐conjugated antibodies targeting CD11c, MHC II, CD80, CD86, CD40, CD200R, CD3, CD4, CD8 and CD25. For regulatory T‐cell identification, Foxp3 intranuclear staining was performed using a Foxp3 staining kit (eBioscience, USA). Fluorescence minus one (FMO) control was employed to establish gating strategies. Flow cytometric analysis was conducted on a BD Canto II flow cytometer (BD, USA), and data processing was performed using FlowJo software (V.10.7.1, USA).

### 2.9 Sample Size

Sample sizes were selected based on previous experience and published studies from our group using established murine models of corneal neovascularisation and high‐risk corneal transplantation. For compound exposure and *Vegfa* mRNA expression analyses, five animals per group were used. For corneal neovascularisation and graft survival experiments, 10 animals per group were included unless otherwise stated.

### 2.10 Statistical Analysis

Data are expressed as mean ± standard deviation (SD). Statistical comparisons were performed using Student's *t*‐test for group differences and the Kaplan–Meier log‐rank test for graft survival analysis. All analyses were conducted using GraphPad Prism (version 8; GraphPad Software, La Jolla, CA, USA). A *p*‐value < 0.05 was considered statistically significant.

## 3 Results

### 3.1 Quantification of Corneal Compound Exposure and *Vegfa* mRNA Expression

In corneal tissue, the highest exposure of VEGF‐A ASO was achieved by 48‐h preincubation of donor corneas, whereas topical application in naïve eyes resulted in moderate ASO uptake (*n* = 5; Figure 2D). A similar distribution pattern was observed with the non‐targeting control ASO (*n* = 5; Figure S1).

In conjunctival tissue, subconjunctival injection consistently resulted in markedly higher ASO accumulation compared with topical eye drop administration, irrespective of corneal suture status or ASO sequence (Figure 2E and Figure S1).

*Vegfa* mRNA expression analysis revealed that preincubation led to the most robust gene suppression in the cornea (*n* = 5; *p* < 0.0001), consistent with its high tissue exposure (Figure 2F). Surprisingly, topical administration in naïve eyes resulted in a significant reduction of *Vegfa* mRNA in both cornea and conjunctiva (*n* = 5; *p* < 0.05), despite the relatively low compound uptake (Figure 2D,E). In contrast, no significant VEGF‐A suppression was observed following subconjunctival injection or suture‐associated eye drop treatment (Figure 2F,G).

### 3.2 Inhibition and Regression of Corneal (Lymph)angiogenesis by Subconjunctival Injection or Topical Eye Drops of VEGF‐A ASO

For assessing the inhibitory effects of VEGF‐A ASO on corneal neovascularisation, subconjunctival injections were administered on Days 0, 2 and 4 following suture placements to induce corneal inflammation and vessel growth. Sutures were removed on Day 7, and subsequent immunofluorescence analyses were performed to evaluate the growth of BVs as well as LVs inhibition (Figure 3A–E). Quantitative analysis demonstrated a statistically significant reduction in both BVs (54.25%, *p* < 0.0001, *n* = 10; Figure 3F) and LVs (64.98%, *p* = 0.0002, *n* = 10; Figure 3G) following VEGF‐A ASO treatment.

**FIGURE 3:**
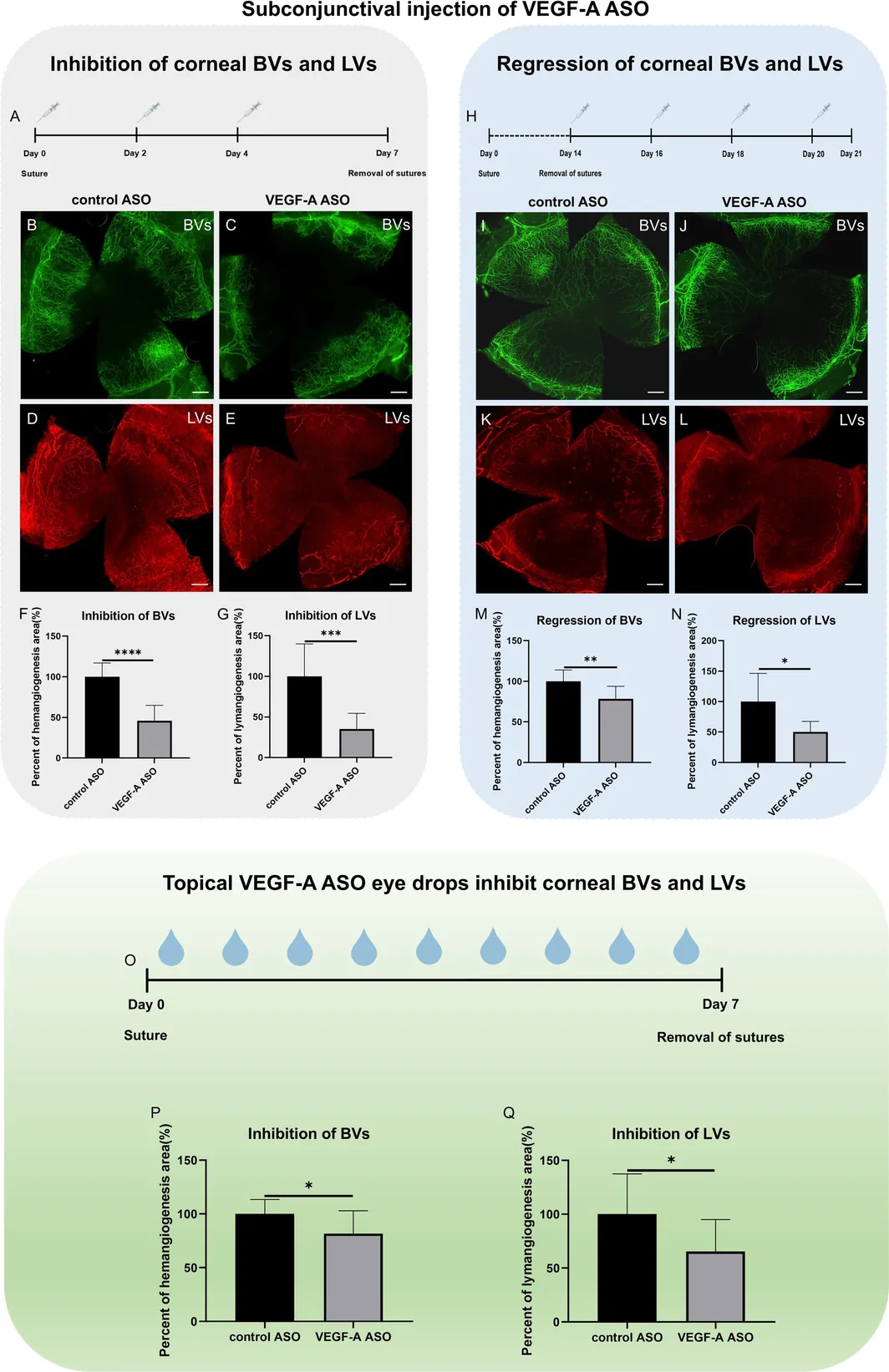
Subconjunctival or topical application of VEGF‐A ASO inhibits and regresses corneal neovascularisation. (A) Protocol for inhibition of corneal blood (BVs) and lymphatic vessels (LVs) in a suture‐induced neovascularisation model via subconjunctival injection. Subconjunctival injections of VEGF‐A ASO or control ASO were administered on Days 0, 2 and 4, with suture removal on Day 7. (B–E) Representative whole‐mount corneal images showing CD31^+^ BVs (green, B–C) and LYVE‐1^+^ LVs (red, D–E) following treatment. (F–G) Quantification of vessel inhibition demonstrated a significant reduction in BVs area (F) and LVs (G) compared to the control group (*n* = 10). (H) Protocol for regression of pre‐established corneal neovascularisation. Following suture removal on Day 14, VEGF‐A ASO or non‐targeting ASO was injected on Days 14, 16, 18 and 20, with corneal analysis on Day 21. (I–L) Whole‐mount staining of CD31^+^ BVs (I–J) and LYVE‐1^+^ LVs (K–L). (M–N) Quantification of vessel regression revealed a significant decrease in BVs (M) and LVs (N) in VEGF‐A ASO‐treated corneas (*n* = 10). (O) Protocol for inhibition of BVs and LVs in a suture‐induced neovascularisation model via topical administration. VEGF‐A ASO or non‐targeting ASO eye drops were administered three times daily for 7 days. (P, Q) BVs and LVs were significantly reduced in the VEGF‐A ASO‐treated group compared to controls (*n* = 10). *p < 0.05, **p < 0.01, ***p < 0.001, ****p < 0.0001. BVs: Blood vessels; LVs: Lymphatic vessels.

To assess the regression of pre‐established corneal vessels, sutures were initially placed to induce neovascularisation and were subsequently removed on Day 14 to allow stabilisation. Subconjunctival injections were administered on Days 14, 16, 18 and 20, and corneal tissues were collected on Day 21 for analysis (Figure 3H–L). Quantification revealed that VEGF‐A ASO treatment significantly enhanced regression of both BVs (21.76%, *p* = 0.0039, *n* = 10; Figure 3M) and LVs (49.75%, *p* = 0.0110, *n* = 10; Figure 3N) compared to the control ASO group.

To assess the inhibitory effects of VEGF‐A ASO eye drops on corneal neovascularisation, treatment was administered three times daily for 7 days in a suture‐induced model, followed by suture removal on Day 7 (Figure 3O). Quantitative analysis revealed a significant reduction in both BVs area (18.35%, *p* = 0.0336, *n* = 10; Figure 3P) and LVs area (34.64%, *p* = 0.0345, *n* = 10; Figure 3Q) in the VEGF‐A ASO‐treated group.

### 3.3 Improvement of Graft Survival in High‐Risk Setting by Subconjunctival Injection of VEGF‐A ASO and Preincubation of Donor Tissue With VEGF‐A ASO

Based on the previous findings, we identified subconjunctival injection and donor preincubation as the most promising application routes for further evaluation in a transplantation model. Subconjunctival injection effectively inhibited both BVs as well as LVs growth and even induced vascular regression, while preincubation of donor tissue led to the most efficient reduction of *Vegfa* mRNA. Given these advantages, we next assessed whether these two strategies could also improve graft survival in a murine high‐risk corneal transplantation setting.

In a high‐risk corneal transplantation model, VEGF‐A ASO or non‐targeting ASO was administered postoperatively every other day for a total of five injections to evaluate its impact on graft survival (Figure 4A). Kaplan–Meier survival analysis demonstrated a significant improvement in graft survival for VEGF‐A ASO treatment compared to controls (*p* = 0.0438, *n* = 10; Figure 4B). Flow cytometric analysis of CD4^+^CD25^+^Foxp3^+^ Tregs in dLNs indicated a significant increase in Treg frequency following VEGF‐A ASO treatment (*p* = 0.0293, *n* = 10; Figure 4C). Additionally, there was no significant difference in corneal BVs as well as LVs and phenotype of DCs in dLNs after transplantation 8 weeks (data not shown).

**FIGURE 4:**
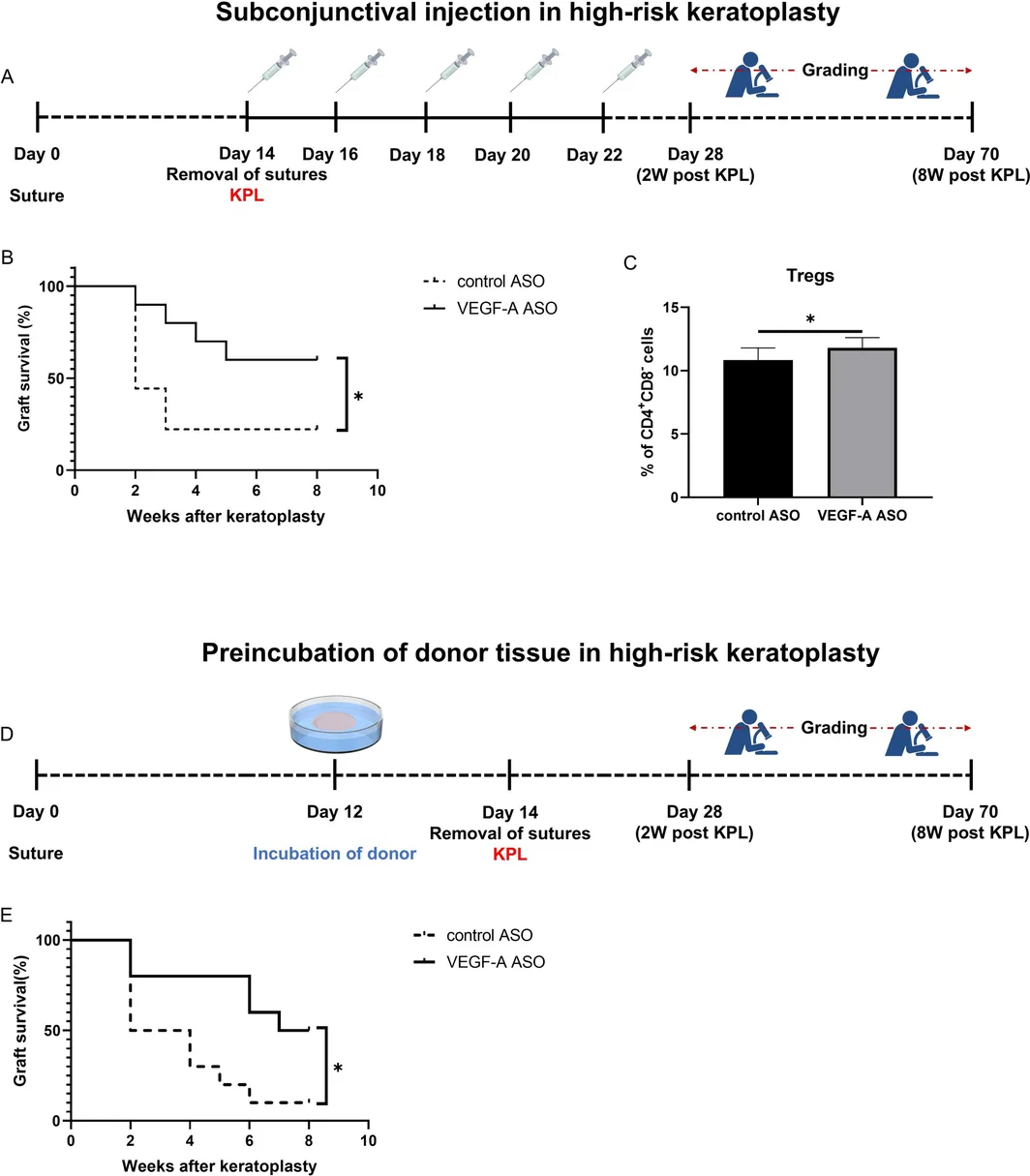
Subconjunctival VEGF‐A ASO or preincubation of donor tissue with VEGF‐A ASO improved the graft survival in high‐risk setting. (A) Protocol for subconjunctival injections in the high‐risk keratoplasty (KPL). (B) Kaplan–Meier survival analysis showed significantly improved graft survival in VEGF‐A ASO‐treated recipients (VEGF‐A ASO, *n* = 10; control ASO, *n* = 9). (C) Subconjunctival administration of VEGF‐A ASO significantly increased the frequency of Treg in dLNs by flow cytometric analysis (VEGF‐A ASO, *n* = 10; control ASO, *n* = 9). (D) Protocol for preincubation of donor tissue in high‐risk KPL. Donor corneas were preincubated with VEGF‐A ASO or non‐targeting ASO for 48 h prior to transplantation. (E) VEGF‐A ASO preincubation significantly prolonged graft survival compared to the non‐targeting ASO group (*n* = 10). *p < 0.05. KPL: Keratoplasty.

Next, donor corneas were preincubated with VEGF‐A ASO or non‐targeting ASO for 48 h before high‐risk corneal transplantation (Figure 4D). Kaplan–Meier survival analysis (Figure 4E) demonstrated a significant improvement in graft survival in recipients of VEGF‐A ASO preincubated donors compared to the non‐targeting ASO group (*p* = 0.0148). There were no further changes in corneal BVs, LVs and phenotype of DCs as well as Tregs in dLNs at 8 weeks post‐keratoplasty (data not shown).

## 4 Discussion

This study analyses for the first time the potential of an ASO targeting *Vegfa* mRNA in corneal tissue against pathologic neovascularisation. It presents two key findings regarding the therapeutic potential of a VEGF‐A ASO in corneal neovascularisation and high‐risk transplantation: (i) First, a VEGF‐A ASO administered via subconjunctival injection or topical eye drops effectively inhibited corneal hemangiogenesis and lymphangiogenesis in vivo. (ii) Second, both postoperative subconjunctival injections and preincubation of donor tissue with a VEGF‐A ASO significantly improved long‐term graft survival in a murine model of high‐risk corneal transplantation.

Corneal neovascularisation is a pathological response to various insults, including congenital defects such as aniridia, hypoxia from contact lenses, trauma, infections and graft rejection, as well as chemical injuries, contributing significantly to vision loss worldwide [15, 26]. A key driver of this pathological process is VEGF‐A, which promotes the formation of new BVs and LVs [24]. Conventional RNAi approaches using siRNA and shRNA target VEGF‐A but require delivery vectors—such as viral carriers or non‐viral systems—to facilitate cellular uptake, introducing limitations like immunogenicity, toxicity and production complexity [11, 27, 28, 29]. In contrast, our study demonstrates that a VEGF‐A ASO can effectively inhibit neovascularisation without the need for carriers, using straightforward approaches such as subconjunctival injections or topical eye drops to suppress blood and lymphatic vessel growth (Figure 3A–G,O–Q). Notably, subconjunctival administration not only prevented vessel formation but also induced regression of pre‐existing vessels, a rare effect in other RNAi‐based therapies (Figure 3H–N).

Previous studies have investigated various gene therapy strategies aimed at improving corneal graft survival by modulating immune responses. Immunomodulatory cytokines such as IL‐10, IL‐4 and IL‐12p40 have been shown to suppress T‐cell activation and shift the immune response from a pro‐inflammatory Th1 profile to an anti‐inflammatory Th2 profile, with IL‐10 showing particular promise in extending graft longevity [30, 31, 32, 33, 34, 35]. Additionally, targeting costimulatory pathways like CD28‐B7 and PD‐L1 reduces T‐cell activation [32, 36], enhancing graft tolerance, while fusion proteins such as CTLA4Ig and ICOSIg have demonstrated the ability to delay rejection [37, 38]. Overexpression of nerve growth factor (NGF) also contributes to prolonged graft survival by promoting corneal wound healing and suppressing immune responses [39]. Furthermore, gene‐modified Tregs and DCs have been shown to foster immune tolerance by suppressing effector T‐cell responses, offering another potential strategy for improving graft acceptance [40, 41, 42, 43]. In addition to these approaches, previous research has demonstrated that blocking inflammatory lymphangiogenesis and the migration of antigen‐presenting cells (APCs) in high‐risk keratoplasty—achieved through systemic or localised depletion of VEGF‐A using monoclonal antibodies—can induce graft tolerance [19, 20, 44]. In this study, we further support these findings by demonstrating that *Vegfa* mRNA depletion by an RNAse H‐recruiting ASO via either subconjunctival injection (Figure 4B) or preincubation of donor tissue (Figure 4E) significantly improves graft survival in high‐risk settings. Our results suggest that temporary inhibition of additional surgery‐induced hemangiogenesis and lymphangiogenesis in already prevascularised high‐risk corneas is sufficient to promote long‐term graft survival. These findings also highlight the importance of the early postoperative period as a critical window for preventing immune sensitisation and reducing the likelihood of rejection.

Our group previously reported that topical application of VEGFR1R2 Trap after high‐risk corneal transplantation led to a reduced frequency of CD40^+^ DCs and an increased frequency of Tregs in the dLNs at 8 weeks post‐transplantation [19]. Additionally, preincubation of donor tissue with VEGFR1R2 Trap 1 day before transplantation resulted in elevated CD200R expression, a marker associated with immune tolerance [20]. In the current study, we observed that subconjunctival injection of VEGF‐A ASO led to a significant increase in Treg frequency in dLNs (Figure 4C). Together, these findings suggest that VEGF‐A blockade may promote immune regulation in regional lymphoid tissue. In addition, we could recently show that anti‐VEGF therapies induce an upregulation of Foxp3 expression in the cornea [45, 46], suggesting that VEGF‐A blockade may also influence the local corneal immune microenvironment. An additional factor that should be considered is the 48‐h ex vivo preincubation of donor corneas before transplantation. Previous studies have shown that organ culture may reduce graft immunogenicity by decreasing the number and viability of donor‐derived antigen‐presenting cells, including DCs, and by reducing the antigenic load within donor tissue [47, 48]. Therefore, preincubation itself may contribute to improved graft survival. However, both experimental groups underwent identical culture conditions, differing only in the presence of VEGF‐A ASO or non‐targeting ASO. Thus, culture‐associated changes in donor immune cell content are unlikely to account for the observed differences between groups. Interestingly, despite the improved graft survival observed following preincubation of donor tissue with VEGF‐A ASO, we found no significant differences in the density of corneal BVs or LVs, nor in immune cell profiles within the dLNs at the 8‐week data not shown. These findings suggest that while preincubation with mRNA‐targeting treatments like a VEGF‐A ASO can initially inhibit angiogenesis and modulate immune responses, these effects may diminish by 8 weeks post‐transplantation. The early immunosuppressive and anti‐angiogenic effects likely contribute to the improved graft survival observed, but over time, the suppression of BVs, LVs and immune cell activation may wane. This outcome is consistent with previous studies where donor tissue served as a drug depot in high‐risk keratoplasty, effectively enhancing graft survival with compounds such as VEGFR1R2 Trap and sCD83 [20, 49].

VEGF‐A contributes not only to pathological hemangiogenesis and lymphangiogenesis but also to physiological tissue repair and epithelial wound‐healing responses [50]. Experimental studies have demonstrated that VEGF signalling promotes corneal epithelial regeneration, whereas topical anti‐VEGF therapy may delay epithelial wound closure in certain settings [51]. Therefore, prolonged or excessive VEGF‐A inhibition may theoretically impair corneal epithelial healing and tissue homeostasis. However, previous studies evaluating topical bevacizumab reported an overall favourable ocular safety profile [52, 53], and no clinically apparent corneal epithelial defects or wound‐healing abnormalities were observed in the present study. Nevertheless, dedicated studies evaluating epithelial wound healing, corneal nerve regeneration and long‐term tissue homeostasis will be important to further establish the safety profile of VEGF‐A ASO therapy before clinical translation.

Comparing the three application routes, we found that the preincubation strategy is the most potent in regards to drug uptake as well as local VEGF mRNA degradation. In these disciplines, the subconjunctival application as well as the eye drops were less effective. This can be due to several reasons. Most obvious is the high accessibility of corneal tissue for the ASO in the preincubation setting. The corneal uptake of the subconjunctival‐injected ASO is limited due to anatomical barriers like the limbus and the sclera. In accordance with this, the conjunctival ASO concentration was the highest by subconjunctival injection (Figure 2E, Figure S1). Similarly, eye drops are limited in access to the corneal stroma, which is nicely demonstrated by the minimal uptake of the ASO by naïve corneas which have an intact epithelial barrier (Figure 2D, Figure S1). In the inflamed corneas, the uptake was already increased (Figure 2D, Figure S1). Furthermore, regarding the different effectivities to degrade VEGF mRNA, one has to take into account that in the preincubation setting the donor cornea cannot be invaded by VEGF mRNA producing immune cells like macrophages whereas in the subconjunctival injection group as well as in the eye drop group, where the inflammation was either induced by the injection itself or by suture placement, respectively. With this inflammation, VEGF mRNA might be replenished constantly. As discussed above, both application routes were still able to inhibit the ingrowth of BVs and LVs (Figure 3). Interestingly, despite low exposure levels (Figure 2D,E, Figure S1), naïve eye drop‐treated eyes showed significant *Vegfa* mRNA reduction in both cornea and conjunctiva (Figure 2F,G), potentially due to reduced baseline transcript turnover in quiescent tissues. Future studies should address cellular uptake dynamics, spatial transcriptomics and immune cell contributions to mRNA turnover in the different modes of action.

A limitation of this study is that the different VEGF‐A ASO delivery approaches were evaluated in separate experiments and were not designed as a direct head‐to‐head comparison. Therefore, although donor cornea preincubation appeared to show a stronger survival benefit, the present study cannot determine whether one administration route is superior to another. Future studies using a predefined comparative design would be required to address this question.

In this context, an interesting direction for future investigation would be the combination of donor cornea pre‐incubation with postoperative topical VEGF‐A ASO administration. Such a peri‐transplant treatment strategy may provide sustained VEGF‐A inhibition throughout the critical phases of graft engraftment and alloimmune activation, thereby potentially enhancing its anti‐(lymph)angiogenic and immunomodulatory effects. However, whether combined administration offers additional benefits over either approach alone remains to be determined experimentally.

In conclusion, this study for the first time demonstrates the significant therapeutic potential of a VEGF‐A ASO in the eye and especially in the cornea in inhibiting and regressing pathologic BVs and LVs. Furthermore, the VEGF‐A ASO treatment enhances outcomes for high‐risk corneal transplantation by effectively inhibiting both corneal hemangiogenesis and lymphangiogenesis. Unlike other RNA‐targeting therapies, a VEGF‐A ASO offers a carrier‐free, targeted and clinically viable approach to prevent graft rejection. These findings lay a strong foundation for future research and clinical applications, positioning VEGF‐A ASOs as a promising therapeutic strategy to modulate pathologic ocular angiogenesis and lymphangiogenesis in various diseases.

## Funding

This work was supported by the German Research Foundation (german: Deutsche Forschungsgesellschaft; DFG) FOR2240, Cu 47/4‐2, Cu 47/6‐1, Cu 47/9‐1 (CC) (BO4489/1‐1, BO4489/1‐2); German Research Foundation Deutsche Forschungsgemeinschaft (DFG) (CRC1607); EU COST Aniridia; EU Horizon 2020 ARREST BLINDNESS; EU Horizon 2020 Restore Vision; Center for Molecular Medicine Cologne, University of Cologne; CECAD Cluster of Excellence, University of Cologne; National Natural Science Foundation of China (82560203).

## Conflicts of Interest

Wei Zhang, Felix Bock and Claus Cursiefen declare that they have no known competing financial interests or personal relationships that could have appeared to influence the work reported in this paper. Christian Weile has been a full‐time employee at Roche Innovation Center Copenhagen A/S while conducting the work for this study. Christoph Ullmer and Sascha Fauser are full‐time employees at Roche Pharma Research & Early Development, Roche Innovation Center Basel.

## Supporting information


**Table S1:** Overview of experimental eye assignment, suture placement and treatment allocation.

**Figure S1:** Tissue exposure of control ASO following different delivery routes.

## Data Availability

Full sequences of VEGF‐A and non‐targeting capture/detection probes are available from the corresponding author or Roche upon reasonable request.
