# Supplementary material for: VEGF‐A Antisense Oligonucleotide Therapy Inhibits Corneal Neovascularisation and Enhances Graft Survival in Murine High‐Risk Corneal Transplantation
Source: Clin Exp Ophthalmol. 2026 Sep 9;54(8):1029–40. doi: 10.1111/ceo.70159 (PMC13741986; doi:10.1111/ceo.70159)
Supplement: Supplementary file 1 [file CEO-54-1029-s002.docx]

Supplementary Table 1. Overview of experimental eye assignment, suture placement, and treatment allocation.

| **Experiment** | **Corresponding figure/panel** | **Treated eye** | **Sutures** | **Intervention** | **Control** |
| --- | --- | --- | --- | --- | --- |
| Subconjunctival injection (exposure & *Vegfa* mRNA) | Fig. 2A | Right eye | Yes | VEGF-A ASO (subconjunctival injection; 3x in 7 days) | Non-targeting ASO (subconjunctival injection; 3x in 7 days) |
| Topical eye drops in naïve eyes (exposure & *Vegfa* mRNA) | Fig. 2B | Right eye | No | VEGF-A ASO (eye drops; 3x/d; 14 days) | Non-targeting ASO (eye drops; 3x/d; 14 days) |
| Topical eye drops in suture-induced eyes (exposure & *Vegfa* mRNA) | Fig. 2B | Right eye | Yes | VEGF-A ASO (eye drops; 3x/d; 14 days) | Non-targeting ASO (eye drops; 3x/d; 14 days) |
| Donor cornea preincubation (exposure & *Vegfa* mRNA) | Fig. 2C | Donor corneal buttons | No | VEGF-A ASO preincubation (once) | Non-targeting ASO preincubation (once) |
| Subconjunctival treatment in the neovascularization inhibition model | Fig. 3A–G | Right eye | Yes | VEGF-A ASO (subconjunctival injection; 3x in 7 days) | Non-targeting ASO (subconjunctival injection; 3x in 7 days) |
| Subconjunctival treatment in the neovascularization regression model | Fig. 3H–N | Right eye | Yes (removed before treatment) | VEGF-A ASO (subconjunctival injection; 4x in 7 days) | Non-targeting ASO (subconjunctival injection; 4x in 7 days) |
| Topical treatment in the neovascularization inhibition model | Fig. 3O–Q | Right eye | Yes | VEGF-A ASO (eye drops; 3x/d; 7 days) | Non-targeting ASO (eye drops; 3x/d; 7 days) |
| High-risk transplantation with postoperative treatment | Fig. 4A–E | Right eye | Yes (pre-transplant) | VEGF-A ASO (postoperative subconjunctival injection; 5x in 10 days) | Non-targeting ASO (postoperative subconjunctival; injection5x in 10 days) |
| High-risk transplantation with donor cornea preincubation | Fig. 5A–E | Right eye | Yes (pre-transplant) | VEGF-A ASO donor preincubation (once) | Non-targeting ASO donor preincubation (once) |
