# Supplementary material for: VEGF‐A Antisense Oligonucleotide Therapy Inhibits Corneal Neovascularisation and Enhances Graft Survival in Murine High‐Risk Corneal Transplantation
Source: Clin Exp Ophthalmol. 2026 Sep 9;54(8):1029–40. doi: 10.1111/ceo.70159 (PMC13741986; doi:10.1111/ceo.70159)
Supplement: Supplementary file 2 [file CEO-54-1029-s001.doc]

**Supplementary Figure 1 Tissue exposure of control ASO following different delivery routes**

(A) Control ASO exposure levels in the cornea after different delivery strategies. The highest corneal ASO levels were observed after 48-hour preincubation, whereas topical application in naïve eyes resulted in the lowest exposure. Subconjunctival injection and topical treatment in suture-induced corneas showed intermediate corneal ASO levels. (B) Control ASO exposure levels in the conjunctiva. Subconjunctival injection led to markedly higher conjunctival ASO accumulation compared with topical eye drop application in both naïve and suture-induced eyes. n=5 per group, *P < 0.05, **P <0.01，***P <0.001，****P < 0.0001.
